# Pulmonary parenchymal involvement caused by *Tropheryma whipplei*

**DOI:** 10.1515/med-2021-0297

**Published:** 2021-06-02

**Authors:** Wen Mei Zhang, Ling Xu

**Affiliations:** Department of Respiratory Medicine, Shanghai Jiao Tong University Affiliated Sixth People’s Hospital, No. 600, YiShan Road, Shanghai, 200233, China

**Keywords:** Whipple’s disease, *Tropheryma whipplei*, pulmonary, cavity

## Abstract

We report a 26-year-old man with left chest pain for 4 days. His chest CT showed a cavity in the left upper lung. Tuberculosis was suspected first, but metagenomics next generation sequencing (mNGS) in bronchoalveolar lavage fluid only detected *Tropheryma whipplei*. *Tropheryma whipplei* is the pathogen of Whipple’s disease. The most frequently involved organs are the eyes, heart, and central nervous system. Pulmonary parenchymal involvement is rare. To our knowledge, this is the first reported case of pulmonary cavity caused by *Tropheryma whipplei*. Nineteen cases of pulmonary parenchymal involvement were found by literature search. The most common respiratory symptom was cough, followed by dyspnea/breathlessness and chest pain. The most common finding in chest imaging was pulmonary nodules, followed by interstitial changes and patchy infiltration. Our case and literature review highlighted that *Tropheryma whipplei* infection should be considered in the differential diagnosis of pulmonary cavity, pulmonary nodules, interstitial changes, and patchy infiltration. mNGS is helpful to improve diagnosis rate.

## 1 Introduction

*Tropheryma whipplei* is the pathogen of Whipple’s disease, which is a rare chronic infectious disease involving multiple systems. Classic Whipple’s disease is more common in middle-aged Caucasian men and extremely rare in the native Asian and African populations [1]. The most frequently involved organs are the eyes, heart, and central nervous system [1]. Pulmonary parenchymal involvement is rare. Little attention has been paid in the published literature to the respiratory features of Whipple’s disease.

## 2 Case report

**Figure 1:**
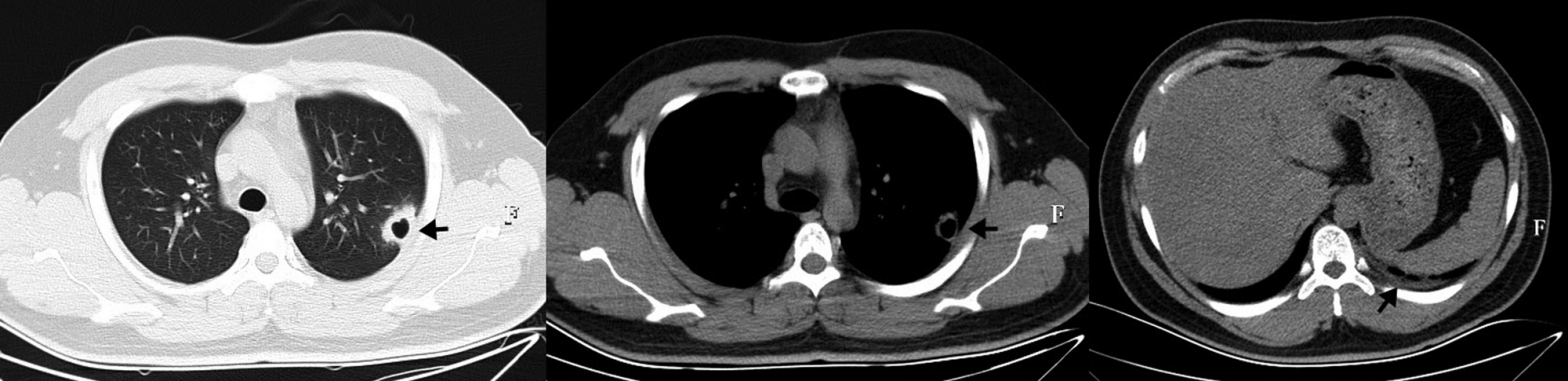
Chest CT before treatment, showing a thick-walled cavity in the left upper lung, with smooth inner and irregular outer margins. The adjacent pleural and the left lower pleural were thickened.

A 26-year-old Chinese male was admitted to our hospital due to breathing-related chest pain on the left for 4 days. He was in good health before, had no immunodeficiency diseases, and did not take any immunosuppressive agents. He was a software engineer, with no history of tourism and no history of contact with patients infected with *Tropheryma whipplei*. Physical examination was normal. The chest CT showed a thick-walled cavity in the left upper lung. Vascular convergence sign and local pleural thickening can be seen (shown in Figure 1). Blood routine test, C-reactive protein, erythrocyte sedimentation rate, procalcitonin, 1,3-β-d-glucan assay, and endotoxin test were normal. Serological tests of *Mycoplasma pneumoniae*, *Chlamydia pneumoniae*, *Legionella pneumoniae,* respiratory viruses, and HIV were negative. Autoantibodies were all negative. Tuberculosis was suspected first, but tuberculin test was negative. In order to determine the cause of the disease, bronchoscopy was performed and showed normal bronchial mucosa. Bronchoalveolar lavage fluid (BALF) was analyzed by metagenomics next generation sequencing (mNGS). *Tropheryma whipplei* was detected and it was the only pathogen identified.

**Figure 2:**
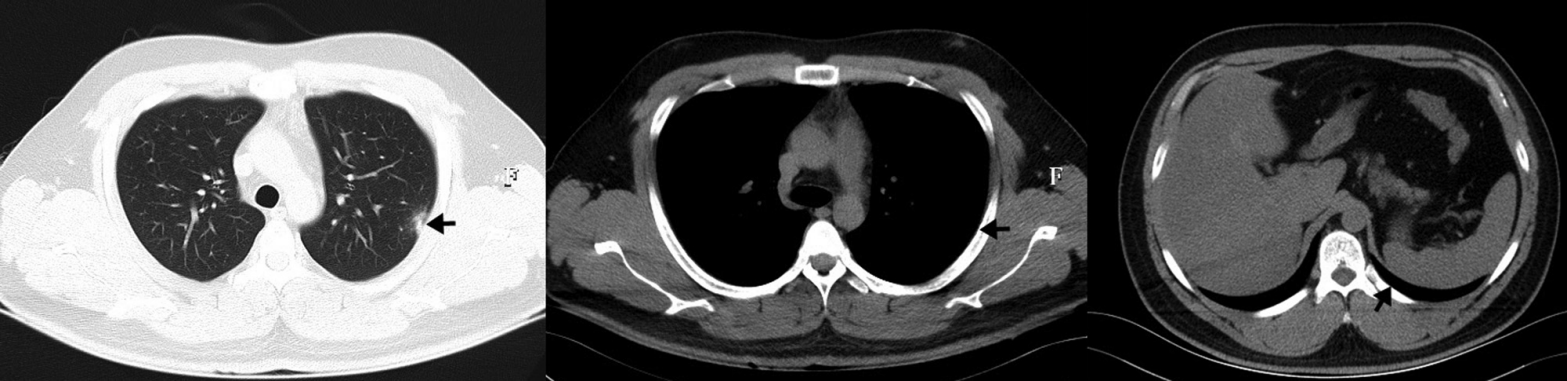
Chest CT after 6 weeks of treatment, showing the cavity disappeared, leaving only a little patchy infiltration. The pleural thickening was improved.


The patient was diagnosed with *Tropheryma whipplei* infection. He was treated with intravenous ceftriaxone (2 g, once daily) for 2 weeks. Then, he received oral administration of trimethoprim–sulfamethoxazole (0.96 g, twice daily). His chest pain was relieved one week after starting treatment. Six weeks after treatment, a follow-up chest CT scan showed that the pulmonary cavity disappeared, leaving only a little patchy infiltration. The pleural thickening was reversed (shown in Figure 2). The patient is still under treatment and follow-up.

**Informed consent:** Informed consent has been obtained from our patient in this study.

## 3 Discussion

Whipple’s disease is a rare chronic infectious disease. It mainly manifests as pleural effusion when the respiratory system is involved, with an incidence of 12.4% [1,2]. In Whipple’s disease, pulmonary parenchymal involvement is rare. Our case is the first diagnosed case of pulmonary cavity caused by *Tropheryma whipplei*. In addition to our case, we identified 19 patients with lung parenchymal involvement in the literature [3,4,5,6,7,8,9,10,11,12,13,14,15,16,17,18]. Their clinical, radiologic, and pathological features were provided in the Table S1.

Among the 20 patients, 13 were male and seven were female. The age at diagnosis ranged from 26 to 81 years old. Respiratory symptoms included cough in 11 cases (55.0%), dyspnea/breathlessness in 10 cases (50.0%), chest pain in five cases (25.0%), expectoration in three cases (15.0%), hemoptysis in one case (5.0%), asymptomatic in two cases (10.0%), and no description in one case. There were 10 patients (50.0%) with arthralgia/arthritis. Among them, four patients had joint symptoms before respiratory symptoms. Diarrhea occurred in 10 patients (50.0%), one before and three after the onset of respiratory symptoms. Other extrapulmonary symptoms included weight loss in 11 cases (55.0%), fever in 11 cases (55.0%), abdominal pain, sweating, and anorexia in three cases (15.0%), respectively. The most common chest radiologic findings were nodules (10/20, 50.0%), which could be solitary or diffuse, ground-glass or solid, including fine nodules or large nodules of several centimeters. The second was interstitial changes (5/20) and patchy infiltration (5/20), accounting for 25.0%, respectively. Cavity was only seen in our case (5.0%). Four cases (20.0%) had hilar/mediastinal lymphadenopathy. Four cases (20.0%) had pleural thickening/adhesions and two cases (10.0%) had pleural effusion. It could be seen that the most common respiratory symptom was cough, followed by dyspnea/breathlessness and chest pain. The chest imaging manifestations were various, including nodules, interstitial changes, patchy infiltration, and cavity.

Although chronic infection is the most common form, acute infection caused by *Tropheryma whipplei* is gradually recognized. Among the 20 patients, there were nine cases of classic Whipple’s disease, one case of atypical Whipple’s disease, five cases of acute pulmonary infection (duration <1 month), and two cases of chronic pulmonary infection (duration >1 month). In the other three patients, the infection was confined to the lung, but the course of disease was unknown. Our results showed that acute lung infection caused by *Tropheryma whipplei* could be manifested as patchy opacity, micronodules, or cavity, while the pulmonary parenchymal involvement caused by Whipple’s disease was mainly manifested as nodules and interstitial changes.

Some studies showed that Whipple’s disease could occur in patients with immunodeficiency or immunosuppression [19]. Immunosuppressive therapy, such as treatment with corticosteroids or tumor necrosis factor antagonists, can worsen Whipple’s disease [1,11]. A study on 176 bronchoalveolar lavage samples showed that more patients in the *Tropheryma whipplei* group were treated with tumor necrosis factor alpha inhibitors compared with the control group, but overall, there was no difference in the immunocompromised status of the patients between the two groups [20]. Of the 20 patients in our study, five had unknown immune status, seven had normal immune status, one had HIV-2 infection, and seven had received immunosuppressive therapy before the diagnosis of *Tropheryma whipplei* infection. Among these seven cases, only one received immunosuppressive therapy due to kidney transplantation for lupus nephritis. The other six cases received immunosuppressive therapy due to misdiagnosis of sarcoidosis (3 cases), arthritis (2 cases), and Behcet’s disease (1 case). Of these six cases, one case deteriorated rapidly after treatment with glucocorticoids, four cases developed diarrhea and weight loss 1–7.5 years after receiving immunosuppressive therapy, and one case developed intermittent fever more than 1 year after receiving immunosuppressive therapy. Therefore, the role of the host’s immune system in the infection of *Tropheryma whipplei* is still largely unresolved. But it is certain that patients with *Tropheryma whipplei* infection should not use immunosuppressants to avoid aggravating the condition.

Before 1991, the diagnosis of Whipple’s disease was based on histopathological examination, characterized by positive periodic acid-Schiff (PAS) stained inclusions within intestinal macrophages. After 1991, PCR was introduced into clinical practice. mNGS is a new technology developed in recent years. Our case was diagnosed by this method. Among the 20 cases, *Tropheryma whipplei* was present in BALF or lung biopsy specimens in 12 cases, three cases were negative for PAS staining, while relevant information was not available in five cases. Pathological examination of lung showed that there was fibrosis in four patients, multiple foam macrophages in four patients, acute fibrinous and organizing pneumonia in one patient, and noncaseating granulomas in one patient. Besides, one patient presented with noncaseating granuloma in mediastinal lymph nodes. Therefore, we speculate that there are two mechanisms of lung parenchymal lesions caused by *Tropheryma whipplei*: (1) inflammatory reaction caused by direct infection of *Tropheryma whipplei* in lung tissues; (2) immune response in lung after infection of *Tropheryma whipplei* in other organs.

Antibiotic therapy is the recommended treatment choice of *Tropheryma whipplei* infection. Due to the high recurrence rate and mortality of Whipple’s disease, long-term treatment is needed. Ceftriaxone or meropenem for 14 days followed by oral co-trimoxazole for 12 months is one of regimens. Some authors suggested that hydroxychloroquine combined with doxycycline is a more rational alternative approach [2]. For acute infections, no evidence has yet been established about treatment duration.

In conclusion, the chest imaging manifestations of pulmonary parenchymal involvement caused by *Tropheryma whipplei* were various. For patients with pulmonary nodules, interstitial changes, patchy infiltration, or cavity, *Tropheryma whipplei* infection should be considered as one of the diseases that need to be differentiated, especially when patients have extrapulmonary symptoms. mNGS is helpful to improve diagnosis rate.

## References

[1] Dolmans RA , Boel CH , Lacle MM , Kusters JG . Clinical manifestations, treatment, and diagnosis of Tropheryma whipplei infections. Clin Microbiol Rev. 2017;30(2):529–55. 10.1128/CMR.00033-16 PMC5355640 28298472

[2] Fenollar F , Puéchal X , Raoult D . Whipple’s disease. N Engl J Med. 2007;356(1):55–66. 10.1056/NEJMra062477 17202456

[3] Pirola RC , Mishkel MA , Macdonald GJ , Liddelow AG . Whipple’s disease. Med J Aust. 1967;2(22):985–8. 10.5694/j.1326-5377.1974.tb93228.x 4170086

[4] Winberg CD , Rose ME , Rappaport H . Whipple’s disease of the Lung. Am J Med. 1978;65(5):873–80. 10.1016/0002-9343(78)90809-4 81613

[5] Cho C , Linscheer WG , Hirschkorn MA , Ashutosh K . Sarcoidlike granulomas as an early manifestation of Whipple’s disease. Gastroenterology. 1984;87(4):941–7. 6205935

[6] Symmons DP , Shepherd AN , Boardman PL , Bacon PA . Pulmonary manifestations of Whipple’s disease. Q J Med. 1985;56(220):497–504. 2413499

[7] Kelly CA , Egan M , Rawlinson J . Whipple’s disease presenting with lung involvement. Thorax. 1996;51(3):343–4. 10.1136/thx.51.3.343 PMC1090658 8779149

[8] Dzirlo L , Hubner M , Müller C , Blaha B , Formann E , Dellinger C , et al. A mimic of sarcoidosis. Lancet. 2007;369(9575):1832. 10.1016/S0140-6736(07)60822-8 17531892

[9] Canessa PA , Pratticò L , Sivori M , Magistrelli P , Fedeli F , Cavazza A , et al. Acute fibrinous and organising pneumonia in Whipple’s disease. Monaldi Arch Chest Dis. 2008;69(4):186–8. 10.4081/monaldi.2008.382 19350842

[10] Nubourgh I , Vandergheynst F , Lefebvre P , Lemy A , Dumarey N , Decaux G . An atypical case of Whipple’s disease: case report and review of the literature. Acta Clin Belg. 2008;63(2):107–11. 10.1179/acb.2008.63.2.009 18575052

[11] Lagier JC , Lepidi H , Raoult D , Fenollar F . Systemic Tropheryma whipplei clinical presentation of 142 patients with infections diagnosed or confirmed in a reference center. Medicine. 2010;89(5):337–45. 10.1097/MD.0b013e3181f204a8 20827111

[12] Fenollar F , Ponge T , La Scola B , Lagier JC , Lefebvre M , Raoult D . First isolation of Tropheryma whipplei from bronchoalveolar fluid and clinical implications. J Infect. 2012;65(3):275–8. 10.1016/j.jinf.2011.11.026 22172770

[13] Urbanski G , Rivereau P , Artru L , Fenollar F , Raoult D , Puéchal X . Whipple disease revealed by lung involvement. a case report and literature review. Chest. 2012;141(6):1595–8. 10.1378/chest.11-1812 22670021

[14] Stein A , Doutchi M , Fenollar F , Raoult D . Tropheryma whipplei pneumonia in a patient with HIV-2 infection. Am J Respir Crit Care Med. 2013;188(8):1036–7. 10.1164/rccm.201304-0692LE 24127807

[15] Chen XL, Sun HL. The imaging manifestations of a case of Whipple’s disease in the lungs. Journal of China-Japan Friendship Hospital. 2015;29(4):263 (Chinese).

[16] Vankeerberghen A , Jonckheere S , De Raeve H , Caluwe R , De Beenhouwer H . Novel Tropheryma species in a lung biopsy sample from a kidney transplant recipient. Clin Microbiol Infect. 2018;24(5):548.e5–8. 10.1016/j.cmi.2017.09.011 28962995

[17] Prudent E , Le Guenno G , Jonckheere S , Vankeerberghen A , Lepidi H , Angelakis E , et al. Fluorescent in situ hybridization can be used as a complementary assay for the diagnosis of Tropheryma whipplei infection. Infection. 2019;47(2):317–21. 10.1007/s15010-018-1243-0 30368732

[18] Li W , Zhang Q , Xu Y , Zhang X , Huang Q , Su Z . Severe pneumonia in adults caused by Tropheryma whipplei and Candida sp. infection: a 2019 case series. BMC Pulm Med. 2021;21(1):29. 10.1186/s12890-020-01384-4 PMC7810182 33451316

[19] Marth T , Raoult D . Whipple’s disease. Lancet. 2003;361(9353):239–46. 10.1016/S0140-6736(03)12274-X 12547551

[20] Lagier JC , Papazian L , Fenollar F , Edouard S , Melenotte C , Laroumagne S , et al. Tropheryma whipplei DNA in bronchoalveolar lavage samples: a case control study. Clin Microbiol Infect. 2016;22(10):875–9. 10.1016/j.cmi.2016.07.010 27432769

